# The European General Practice Research Network Presents the Translations of Its Comprehensive Definition of Multimorbidity in Family Medicine in Ten European Languages

**DOI:** 10.1371/journal.pone.0115796

**Published:** 2015-01-21

**Authors:** Jean Yves Le Reste, Patrice Nabbe, Charles Rivet, Charilaos Lygidakis, Christa Doerr, Slawomir Czachowski, Heidrun Lingner, Stella Argyriadou, Djurdjica Lazic, Radost Assenova, Melida Hasaganic, Miquel Angel Munoz, Hans Thulesius, Bernard Le Floch, Jeremy Derriennic, Agnieska Sowinska, Harm Van Marwijk, Claire Lietard, Paul Van Royen

**Affiliations:** 1 Department of General Practice, Université de Bretagne occidentale, Brest, France; 2 Associazione Italiana Medici di Famiglia (AIMEF), Bologna, Italy; 3 Allgemein Medizin Hochschule Göttingen, Göttingen, Germany; 4 Department of Family Doctor, University Nicolaus Copernicus, Torun, Poland; 5 Allgemein Medizin Hochschule Hannover, Hannover, Germany; 6 The Greek Association of General Practitioners (ELEGEIA), Thessaloniki, Greece; 7 University of Zagreb, School of Medicine, Andrija Stampar School of Public Health, Department of Family Medicine, Zagreb, Croatia; 8 Department of General Practice, University of Plovdiv, Plovdiv, Bulgaria; 9 Department of General Practice, University of Sarajevo, Sarajevo, Bosnia; 10 IDIAP Jordi Gol, Unitat de Support a La Recerca, Barcelona, Spain; 11 Department of Clinical Sciences, Lund University, Malmo, Sweden; 12 Department of English, Nicolaus Copernicus University, Torun, Poland; 13 Department of General Practice, VU University Medical Center, Amsterdam, The Nederland; 14 Department of Public Health, Université de Bretagne occidentale, Brest, France; 15 Department of Primary and Interdisciplinary Care, Faculty of Medicine and Health Sciences, Universiteit Antwerpen, Antwerpen, Belgium; University of Modena and Reggio Emilia, ITALY

## Abstract

**Background:**

Multimorbidity, according to the World Health Organization, exists when there are two or more chronic conditions in one patient. This definition seems inaccurate for the holistic approach to Family Medicine (FM) and long-term care. To avoid this pitfall the European General Practitioners Research Network (EGPRN) designed a comprehensive definition of multimorbidity using a systematic literature review.

**Objective:**

To translate that English definition into European languages and to validate the semantic, conceptual and cultural homogeneity of the translations for further research.

**Method:**

Forward translation of the EGPRN’s definition of multimorbidity followed by a Delphi consensus procedure assessment, a backward translation and a cultural check with all teams to ensure the homogeneity of the translations in their national context. Consensus was defined as 70% of the scores being higher than 6. Delphi rounds were repeated in each country until a consensus was reached

**Results:**

229 European medical expert FPs participated in the study. Ten consensual translations of the EGPRN comprehensive definition of multimorbidity were achieved.

**Conclusion:**

A comprehensive definition of multimorbidity is now available in English and ten European languages for further collaborative research in FM and long-term care.

## Introduction

The concept of multimorbidity was first published in 1976 [1] in Germany and remained almost entirely restricted to German publications for 14 years. Between 1976 and 1990 only 72 articles used the term multimorbidity in their text, of which 66 were written in German. In 1990 the concept became internationally recognized through research [2].

The concept of multimorbidity was an addition to the concept of comorbidity. Comorbidity was defined as any disease or risk factors that could interact with one main disease with the effect of making it worse [3–5]. Multimorbidity has been defined by the World Health Organization (WHO) as people being affected by two or more chronic health conditions [6]. The intention of the WHO was to look at all conditions in one individual that could impact on that individual’s global health status. However the word ‘condition’ was not sufficiently clear for practical purposes (for instance, whether a treated disease was a ‘condition’ in this sense), and could lead to numerous interpretations.

Multimorbidity is a very interesting and challenging concept particularly for Family Medicine and long term care, given the increasing prevalence of chronic illness in an aging population across all developed countries. It is closely related to a global or comprehensive view of the patient, which is a core competency of Family Medicine (FM), as defined for instance by the World Organization of National Colleges, Academies and Academic Associations of General Practitioners/Family Physicians (WONCA) [7]. It is a global ‘functional’ view (useful for Long-Term Care) versus a ‘disease’ centered point of view (useful for acute care) [8]. It is also a very interesting concept, when applied to patients, as it gives a global overview of all the factors that could lead to frailty [9–10]. Frailty is a new concept, formulated to help physicians identify decompensating patients. Its link with multimorbidity has already been discussed [11] and a call to action for a consensus on Frailty has been formulated[12].

The European General Practice Research Network (EGPRN) is committed to concepts that could advance research in primary care throughout Europe. The EGPRN has created a research agenda specifically designed for methodological and instrumental research, which includes the development of primary care epidemiology, focusing on patient-centered health [13]. A clear definition of the concept of multimorbidity (i.e. one which is both understandable and usable for further collaborative research) is an important objective for a research network of this type. It will help researchers in FM to investigate the complexity of patients’ conditions and their overall impact on patients’ health. This definition of multimorbidity could be an additional tool for Family Physicians (FPs), enabling them to identify frail patients and prevent decompensation.

A research team, including 9 national groups, all active within the EGPRN, has created a research community for the purpose of clarifying the concept of multimorbidity for FM throughout Europe [14]. An initial review, presented in an EGPRN meeting in spring 2011 [15], identified more than one hundred different definitions used by academic researchers. Such a large number of definitions added more confusion than clarification to the discussion. It led the group to the production of a comprehensive definition of multimorbidity through a systematic review of literature, in which all multimorbidity criteria were scanned and classified by theme [16]. That comprehensive definition of multimorbidity was published after a careful check of its wording and meaning by a working group of three MD researchers from the Irish College of General Practitioners, an MD researcher from the Malta College of Family Doctors and two native English speaking official translators from the University of Brest (France). [16]

This definition had then to be translated into most European languages for use in further collaborative research. It has been previously demonstrated that translating definitions, index or scales is a risky task in medical science[17]. The challenge is to establish a cultural homogeneity between the translations in order to ensure a qualitative transfer of content and that task is as difficult in the medical sciences as it is in literature[18]. The purpose of this research was to translate the exhaustive definition of multimorbidity into ten European languages with the help of a multinational team, with the objective of maintaining a strong homogeneity across those translations.

## Materials and Methods

Maintaining homogeneity between translations needed a cautious and step by step method [19–21]. For all participating countries (Bosnia, Bulgaria, Croatia, France, Germany, Greece, Italy, Poland, Spain) the forward and backward translation of the original English definition has been assessed using a Delphi consensus procedure [22].

### Ethics Statement

The study was approved by the ethics committee of the University de Bretagne Occidentale. The participants provided their written informed consent to participate in the study. The ethics committee approved the consent procedure.

### Research team

First, a research team (including several medical researchers and official translators for each country) was asked to translate the definition from English into their native language. For Spain and Catalonia, a double-language team was used as Catalan is a regional language of Spain.

### Participant selection

The next stage was to send the English multimorbidity definition and its native translation by email to a group of at least 10 and, if possible, 30 national expert FPs. Variations in the sample size were due to the limited number of such experts in small countries and the need to have a larger purposive sample, if possible, allowing a less tentative interpretation of results. As the Delphi technique is a qualitative method, the samples needed to be comparable in terms of homogeneity but not in terms of representativeness. Nevertheless, the Delphi technique is valid, in terms if its effect on outcomes, irrespective of sample size, [23–24]. The study’s scientific committee stressed the role of gender, experience, age, level of English and research or teaching activities to determine their selection. Each participant was contacted separately using emails to avoid contamination, according to the methodology for the Delphi procedure [24].

### Data collection

From May 2012 to December 2012 all experts were then asked to assess the equivalence of the translations on a scale from 1 (absolutely no agreement) to 9 (full agreement) and had to write down their remarks and opinions for each translation ranked below 7. Consensus was defined as at least 70% of the participants rating the consensual definition at 7 or above. This definition of consensus in a Delphi round is the strongest possible definition, according to the Delphi methods, and the RAND UCLA method that is a modified Delphi technique [25]. The RAND UCLA is accepted as a strong formal consensus methodology by Health agencies, such as the NHS in the UK and the Agency for Healthcare Research and Quality in the US[25–26]. This process, called a Delphi round, had to go on until a consensus was achieved. Between each round all discordances had to be taken into account. All suggestions and remarks made by the experts were incorporated into the translation with the objective of defining a new version for the next round. Once the consensual definition in the native language had been established, two other translators did a backward translation from the native language into English.

### Data Analysis

These English consensual back translations had to be examined in order to ensure their semantic and conceptual homogeneity by the study’s scientific committee which consisted of four professors of Family Medicine, one associate professor in public health and one associate professor in Family Medicine drawn from Belgium, France and The Netherlands. The national origins of these researchers could have influenced the outcomes. However, this possible bias was limited as all were experienced researchers and published authors drawn from various countries, in order to ensure heterogeneity, as well as experts in scientific and medical English. Changes could be instigated at that point, depending on the advice of the scientific committee.

To ensure cultural homogeneity, they were then analysed by the research group which undertook a cultural check [27–28]. It was an iterative procedure including a physical meeting in May 2013 during the EGPRN meeting in Kusadasi (Turkey) and exchanges by e-mail before and after the meeting to prepare data and validate the results. The group was composed of all the team leaders and an English linguist from the University of Torun (Poland). English speaking countries had no role to play in the translation into the other European languages. English native speakers were, however, essential to the checking process which followed the backward translations. As a possible loss of meaning could have occurred, the wording and meanings of those backward translations were double-checked by using two native English speakers, official translators from the University of Brest (France), before the cultural check took place. The cultural check needed to take into account that some language conventions (affirmative or passive voice, for example) could express the same meaning within two languages. It had to be very cautious about:

- The control of the study quality throughout the follow-up to the research process which was confirmed by the national team’s leaders and the scientific committee of the study
- The decision to look carefully at changes in meaning and especially at concepts within the translations using tables to help comparison between translations
- The control of the quality of each final translation as the expression of all the concepts in the original language, using tables to record discordances and each participant’s comment.
- The synthesis of all the translations in order to compare them. Their presentation to the research group used tables for all translations, all changes and all comments.

Depending on the result of the cultural check, some changes in the definition’s phrasing were undertaken to ensure homogeneity within the definitions. As no English speaking country was involved in the research process for the English consensual back translations, the wording and meanings were double-checked by using two native English speakers, official translators from the University of Brest (France) after the cultural check. Then all final translations and their backward translations were sent for agreement to the study’s scientific committee.

## Results

### Sample

#### Participants

The nine teams in the different countries consisted of 12 to 30 members. In total there was a good gender distribution, having a mean age of 48 years and on average 18 years of practice experience. All team members had reasonable experience of English usage (in speech, reading and writing). The number of their publications in English averaged 5,91. (See Table 1).

**Table 1:** Expert Panel Characteristics.

	**COUNTRY**	**Gender**		**Average Age in years**	**Average years of practice**	**Competence in English**			**Average English publications**	**Other publications**
	**Total N = 229**	**M**	**F**			**Read**	**Spoken**	**Written**		
1	Bosnia N = 14	5	9	43,29	16,71	All	All	All	2,79	8,43
2	Bulgaria N = 30	11	19	47,03	21,8	All	All	All	0,27	1
3	Croatia N = 23	3	20	50,13	23,43	All	All	All	14,57	51,3
4	France N = 30	18	12	47,43	19,17	All	All	All	3,23	16,57
5	Germany N = 30	21	9	56,46	18,97	All	All	All	1,5	6,37
6	Greece N = 30	18	12	45,67	12,63	All	All	All	10,2	61,22
7	Italy N = 30	19	11	50,7	24,17	All	All	All	4,38	19
8	Poland N = 30	15	15	43,67	12,2	All	All	All	1,75	6,27
9	Spain N = 12	8	4	48,33	22,58	All	All	All	15,33	6
Global Average		50,69%	49,31%	48,26	18,82	100%			5,91	20,45

#### Number of rounds

Countries needed one to two Delphi rounds to achieve their translations. When two rounds were needed, it was mainly the result of experts’ confusion. In those countries the experts thought they could discuss the definition itself. After a formal explanation of their task, the rounds were successful. Even where all the consensus scores were high, the lower they were, the more comments were expressed, as expected by this method. Comments were numerous expressing the richness of the exchanges. (See Table 2).

**Table 2:** Number of Delphi Rounds and Number of Comments in Each Country.

**Country**	**Number of Delphi rounds**	**Mean consensus score for final Round**	**Number of score>6 as percentage**	**Total number of comments**
Bosnia	2	7,8	100%	27
Bulgaria	1	8,2	96,67%	6
Croatia	1	8,5	100,00%	7
France	2	7,4	80.00%	63
Germany	2	7,8	81,00%	23
Greece	1	8,3	100,00%	6
Italy	1	7,6	80.00%	18
Poland	1	7,56	83.33%	9
Spain	1	7,08	75.00%	12
Catalonia	1	7,25	75.00%	12
Mean for Europe	1.3	7,895	96%	18,3

### Analysis

#### Challenged terms

The terms which were challenged the most within Europe during translation were:

- Social network
- Burden of disease
- Health care consumption
- Modifiers
- Health outcomes
- Frailty
- Biopsychosocial factors

Grammatical rewording suggestions were frequent. All comments were carefully recorded, even where consensus had been obtained, in order to help the cultural check.

#### Backward translation

Backward translations were finalized and sent to the original authors of the definition. The authors validated the translated definitions using the backward translations to check that there were no semantic or conceptual changes in comparison with the original English definition.

#### Cultural check

The final phase was the cultural check to ensure the transculturality and homogeneity of the translated definitions.

For Bosnia, the translated definition was not different from the original one despite the fact that the phrases often involved the inversion of subject and complement. Some articles were added to the original definition (“a chronic disease” instead of “chronic disease”) with a little more stress placed on the presence of one chronic disease in a multimorbid patient. The group concluded that there was no change in meaning.

For Bulgaria, some articles were added to the original translation (“a chronic disease” instead of “chronic disease”) with the same meaning as the Bosnian changes. The group concluded that there was no change in meaning. There was a change concerning ‘connection’ instead of ‘association’ (bio psychosocial factors “connected or not with the disease” instead of “associated or not with the disease”). But there is only one word in Bulgarian to express those two meanings and the group concluded that there was no change of meaning. The “somatic risk factors” were changed to “risk factors” as risk factors are always understood as somatic by Bulgarian FPs. The Bulgarians changed ‘network’ into ‘social network’, when describing the patient’s environment, to be sure the concept was as broad as in the original English definition. In this way, they encompassed, in Bulgarian, not only family and friends (which is the meaning of network in Bulgarian) but also the social infrastructures surrounding the patient, as was intended in English. They modified « may modify the health outcomes » to “Multimorbidity can lead to a change in the health outcomes”. This phrasing is less emphatic than the original. Nevertheless, the research group did not think that the meaning was radically changed and kept the Bulgarian version.

For Catalonia there was the same difference as in Bulgaria, regarding the use of articles. There was no difference in meaning.

For Croatia, the “somatic” risk factor was present in the first sentence of the definition but not in the second. The explanation was different from Bulgarian, as the Croatians did not want to repeat the same item twice, seeing it as an underlying factor. All the articles in the second paragraph were omitted as the Croatians wanted to simplify the definition, the way it should be in their language. The group concluded that there were no differences in meaning.

For France, there was the same difference as in Bulgaria, regarding the use of articles, and turning the second paragraph the other way round, with the same explanations. “The effects of multimorbidity may be modified by” instead of “may function as modifiers (of the effects of Multimorbidity).” There was no difference in meaning.

For Germany, there were some significant changes as the backward translation did not reflect the German version. As an example, the word ‘condition’ appears in the first sentence of the backward translation and “Erkrankung” which means ‘disease’ is the only one used in the German version. The back translation was corrected by another team of linguists and the only difference was the affirmative phrasing in German with no use of the conditional tense. This loss of the conditional tense is cultural in spoken German so this was accepted because this definition is intended to be understood by everyone, including patients. There was a final difference between “reduced quality of life” instead of “decreased quality of life” which seemed unchanged in meaning for the research group.

For Greece, there were many differences in relation to an affirmative phrasing in the Greek language (even more so than in German) with the use of “can” instead of “may” which was accepted as it there is no difference in meaning in Modern Greek. The “health service use” occurred instead of the “health service consumption” due to the fact that in Greek the word consumption has the meaning of spending or expenditure and was better encompassed by “use”.

For Italy, the use of “can be defined” instead of “is defined” comes from the fact that Italians did not use “is defined”, preferring to express this idea by using ‘can be defined’ or “may be defined” with the verb “potere” (being able to). The use of “chronic illness” instead of “chronic disease” came from the point that “malattia” in Italian carries both meanings. The same difficulty with the use of articles was observed as in Bulgaria and in France. The word “ogni” in Italian could be translated as ‘any’ or ‘every,’ with no change in meaning, and defines a more global point of view which does not change the meaning of the sentence. A “worsening quality of life” occurred instead of a “decreased quality of life” which encompassed a more affirmative idea or greater fear about multimorbidity in Italy. This seems to be due to the greater presence of multimorbidity in Italian practice which leads to a more active phrasing.

For Poland, the same difference with the use of articles was observed as in Bulgaria, France and Italy. The use of “related” instead of “associated” looked stronger but did not change the meaning and was accepted. There was the same difficulty with ‘risk factor’ instead of ‘somatic risk factors’ as in Bulgaria, with the same underlying meaning pointing to the same conclusion. The “use of health care services” replaced ‘health care consumption,” as in Greece, but for a different reason which is the lack of available medication, an additional factor in health services in Poland. The Polish translators forgot the second part of the sentence at the end of the second paragraph (‘of the effects of multimorbidity’) but this was added in the final definition. Then ‘weakness’ replaced ‘frailty’ as there is only one word for the two concepts in Polish.

For Spain and Catalonia there was the same difference as in Bulgaria, regarding the use of articles. There was no difference in meaning.

The necessary changes were integrated into the final definitions and proposed to the study’s scientific committee. The committee found no semantic, conceptual or cultural changes compared with the original definition and so the translations obtained were validated for all the countries concerned. (See Table 3)

**Table 3:** English Original and final translation for each country.

**English original**	**Bosnia**	**Bulgaria**	**Croatia**
Multimorbidity is defined as any combination of chronic disease with at least one other disease (acute or chronic) or bio-psychosocial factor (associated or not) or somatic risk factor. Any bio-psychosocial factor, any somatic risk factor, the social network, the burden of diseases, the health care consumption and the patient’s coping strategies may function as modifiers (of the effects of Multimorbidity). Multimorbidity may modify the health outcomes and lead to an increased disability or a decreased quality of life or frailty.	Multimorbidnost(pacijent sa više bolesti u isto vrijeme) je definisana kao svaka kombinacija bolesti sa najmanje još jednom nekom bolešću(akutnom ili hroničnom) ili bio-psihosocijalnim faktorom koji je udružen ili ne) ili somatskim faktorom rizika. Svaki bio-psiho-socijalni faktor,svaki faktor rizika,socijalna podrška,raširenost bolesti,korištenje zdravstvene zaštite i način kako se sam pacijent nosi sa bolešću,može dovesti do promjene.(efekata multimorbidnosti). Multimorbidnost-višebolesnost može mijenjati ishode zdravlja i voditi povećanoj nesposobnosti ili sniženom kvalitetu života ili povećanoj osjetljivosti.	Полиморбидност се определя като всяка комбинация от хронично заболяване, с поне едно друго заболяване (остро или хронично) или свързан или не със заболяването био-психо-социален фактор или друг соматичен рисков фактор. Всеки био-психо-социален фактор, всеки рисков фактор, социалната среда, тежестта на заболяванията, използването на здравни услуги и стратегии на пациента за справяне могат да оказват влияние върху ефектите на полиморбидността. Полиморбидността може да доведе до промяна на очакваните резултати и до по-висока степен на инвалидност, понижено качество на живот или слабост.	Multimorbiditet označava bilo koju kombinaciju kronične bolesti s barem još jednom bolesti (akutnom ili kroničnom), ili s biopsihosocijalnim čimbenikom (pridruženim ili nepridruženim) ili sa somatskim čimbenikom rizika. Bilo koji biopsihosocijalni čimbenik, bilo koji čimbenik rizika, društveno okruženje, teret bolesti, korištenje zdravstvene zaštite te načini bolesnikova nošenja s bolešću, mogu djelovati kao modifikatori (na učinke multimorbiditeta). Multimorbiditet može utjecati na zdravstvene ishode te dovesti do povećanja nesposobnosti ili do smanjenja kvalitete života ili do nemoći.
			
France	Germany	Greece	Italy
La multimorbidité est définie comme toute combinaison d’une maladie chronique avec au moins: une autre maladie (aiguë ou chronique) ou un facteur biopsychosocial (associé ou non) ou un facteur de risque somatique. Les effets de la multimorbidité peuvent être modifiés par: tout facteur biopsychosocial, tout facteur de risque somatique, le réseau social, le poids des maladies, la consommation de soins de santé et les stratégies adaptatives du patient. La multimorbidité peut modifier les résultats de santé et mener à une augmentation du handicap ou à une diminution de la qualité de vie ou à la fragilité.»	Definiert als jegliche Kombination einer chronischen Erkrankung mit zumindest einer weiteren Erkrankung (akut oder chronisch), oder einem bio-psycho-sozialen Faktor(assoziiert oder nicht) oder einem somatischen Risikofaktor. Jeglicher bio-psycho-soziale Faktor, jeglicher Risikofaktor, das soziale Netzwerk, die Krankheitslast, die Inanspruchnahme des Gesundheitssystems sowie persönliche Bewältigungsstrategien können die Auswirkungen von Multimorbidität beeinflussen. Multimorbidität kann Gesundheitsparameter beeinflussen und Funktionseinbußen verstärken. Sie kann auch die Lebensqualität reduzieren oder zu Gebrechlichkeit führen.	Ως πολυνοσσηρότητα ορίζεται κάθε συνδιασμός οξέων ή χρόνιων νοσημάτων με ή χωρίς συσχετιζόμενους ή μη συσχετιζόμενους βιοψυχοκοινωνικούς παράγοντες ή σωματικούς παράγοντες κινδύνου. Αυτοί οι παράγοντες μπορούν επίσης να λειτουργήσουν ως τροποποιητές, παράλληλα με τον κοινωνικό ιστό, τη χρήση υπηρεσιών υγείας και τις στρατηγικές αντιμετώπισης του ασθενούς. Μπορεί να τροποποιήσει τα αποτελέσματα στην υγεία και να οδηγήσει σε μια αυξημένη ανικανότητα, μια μειωμένη ποιότητα ζωής ή ευθραστότητα.	Si definisce multimorbidità ogni combinazione di una malattia cronica con almeno un’altra malattia (acuta o cronica), o un fattore bio-psicosociale (associato o meno), o un fattore di rischio somatico. Ogni fattore bio-psicosociale, ogni fattore di rischio somatico, la rete sociale, il carico delle malattie, l’uso dei servizi sanitari e le strategie con cui i pazienti affrontano i loro problemi possono fungere da agenti modificanti (degli effetti di multimorbidità). La multimorbidità può modificare i risultati di salute e portare ad un incremento della disabilità o ad un peggioramento della qualità della vita o a fragilità.
			
Poland	Spain (Castilian)	Spain (Catalan)	
Wielochorobowość jest definiowana jako jakiekolwiek połączenie choroby przewlekłej z przynajmniej jeszcze jedną chorobą (ostrą lub przewlekłą) lub z czynnikami bio-psycho-społecznymi (związanymi z nią lub nie) lub z czynnikami ryzyka. Jakikolwiek czynnik bio-psycho-społeczny, czynnik ryzyka, sieć społeczna, obciążenie chorobami, korzystanie z opieki zdrowotnej i strategie radzenia sobie przez pacjenta mogą funkcjonować jako modyfikatory. Wielochorobowość może modyfikować wyniki zdrowotne i prowadzić do zwiększonej niepełnosprawności lub obniżenia jakości życia lub osłabienia.	Se define multimorbilidad como cualquier combinación de una enfermedad crónica con al menos otra enfermedad (aguda o crónica) o un factor biopsicosocial (asociado o no) o un factor de riesgo. Cualquier determinante bio-psicosocial, cualquier factor de riesgo, la red social, la carga producida por las enfermedades, el uso de recursos sanitarios y las estrategias de afrontamiento del paciente pueden actuarcomo modificadores del efectos de la multimorbilidad. La multimorbilidad puede modificar los resultados en salud y conducir a una mayor discapacidad o una menor calidad de vida o fragilidad.	Es defineix multimorbiditat com qualsevol combinació d’una malaltia crònica amb com a mínim una altra malaltia (aguda o crònica) o un determinant biopsicosocial (associat o no) o un factor de risc. Qualsevol determinant psicosocial, qualsevol factor de risc, la xarxa social, la càrrega generada per les malalties, l’ús de recursos sanitaris i les estratègies d’afrontament del pacient poden funcionar com a modificadors dels efectes de multimorbiditat. La multimorbiditat pot modificar els resultats en salut i conduir cap a una major discapacitat o una menor qualitat de vida o fragilitat.	

## Discussion

### Main Results

These studies are a consecutive stage of the EGPRN project, which aims to provide a comprehensive definition of Multimorbidity throughout Europe [16]. The main findings are the translations of the English definition of multimorbidity into ten European languages (Table 3). The homogeneity of the translations is of importance for further collaborative research within EGPRN. The homogeneity of the translations has been evaluated in a semantic, conceptual and cultural way which confirms that these translations make provision for the cultural background in which FPs cope with problems in their practices, and demand a holistic approach to the patient.

### Strengths and limitations of the study

The Delphi technique for translation had its own strengths and weaknesses. Nevertheless, it is seen as an accurate consensus technique in health research [29–30]. It has also been used for consensus on care processes [31], patient safety measures [32] and detection and referral for pathologies [33]. There was no information bias in this study as all data was sent to all experts and group members. There was no selection bias either. Even though the scientific committee was concerned about the small size of the Bosnian and Spanish-Catalan groups, it was reassured by the homogeneity of their definitions, which were also the most obvious. In some countries (France and Germany), during the first round, some of the participants believed they had to evaluate the accuracy of the definition which led to a confusion bias. This bias was disentangled at the beginning of the second round while emphasizing the role of translation as the only goal of the study. The sample’s characteristics were very carefully followed up in every country to ensure that the participants were genuine experts, both in Family Medicine and in use of English.

### Key points

A standardized and reproducible definition of multimorbidity is of importance in developed countries where a larger proportion of the population is elderly. This comprehensive definition is helpful for targeting resources in a far more accurate way than the WHO definition [6]. In addition, it gives more focused prognoses for individuals and improves risk management. It improves clinical decision making, in terms of risk/benefit evaluation. It could help decision-making when considering the position of an individual on the spectrum of palliative versus aggressive care.

When considering the previous definitions, most authors agreed to reject any concept which was too vague or insufficiently discriminating for the selection of patients with the diseases mentioned. Those caused problems of interpretation, inclusion of patients, induced a lack of power and confounding factors [34–37]. This comprehensive definition and its translation into ten European languages encompasses all definitions of multimorbidity found in literature [16] and will override previous limitations.

The holistic approach and the patient centered care core competencies of Family Medicine, according to WONCA, [7] promote a concept of multimorbidity which is closer to the result of this study than any other.

### Implications for practice and future research

The purposes of a standardized and reproducible definition of multimorbidity are numerous and its translation into ten European languages is of great value for further research. A more comprehensive and homogeneous definition leads to better focused research, especially for quality of care and cost of care. This study is included in an EGPRN project, which aims to define the best possible intervention to prevent depression in multimorbid patients. For inclusion a comprehensive and homogeneous definition of multimorbidity within 11 European languages (including English) was essential.

## Conclusion

This study has finalized, through a careful forward backward translation, including a Delphi consensus process, ten European, homogeneous translations of the published English Multimorbidity definition from the EGPRN. In the light of an increasing number of elderly patients across Europe, [38–39] introducing these translations and their semantic, conceptual and cultural homogeneity, was a necessary and a relevant step, especially for further research.

The implementation of the new definition is intended to help European FPs to identify multimorbid patients. It could also be of importance to other Long-Term Care Physicians (geriatricians for example), as well as policy makers, to plan an optimal management of patients, and to lower the burden of multimorbidity [40].

The European translations enable the EGPRN research team to proceed to the next step, which is qualitative research, in order to find the value added by FPs to the concept of multimorbidity. This will be achieved by using a grounded theory analysis and a deductive analysis from the translated definitions of multimorbidity. Then the study’s scientific committee will be able to discuss which means could be used to ensure the implementations of Multimorbidity into databases and registers. Eventually an International Classification Primary Care code will be put forward to the ICPC committee of the WONCA [41].
